# Nutritional Enhancement of Farmed Salmon Meat via Non-GMO Nannochloropsis Gaditana: Eicosapentaenoic Acid (EPA, 20:5 n-3), Docosapentaenoic Acid (DPA, 22:5 n-3) and Vitamin D3 for Human Health

**DOI:** 10.3390/molecules25204615

**Published:** 2020-10-10

**Authors:** Ivonne Lozano-Muñoz, Susana Muñoz, Nelson F. Díaz, Alberto Medina, Jazmín Bazaes, Carlos Riquelme

**Affiliations:** 1 Departamento de Producción Animal, Facultad de Ciencias Agronómicas, Universidad de Chile, Santiago 8820000, Chile; smunoz@uchile.cl (S.M.); nelsond@uchile.cl (N.F.D.); 2 Departamento de Acuicultura y Recursos Agroalimentarios, Universidad de Los Lagos, Osorno 5290000, Chile; amedina@ulagos.cl; 3 Centro de Bioinnovación, Facultad de Ciencias del Mar y Recursos Biológicos, Universidad de Antofagasta, Antofagasta 1240000, Chile; jazminbazaes@gmail.com (J.B.); carlos.riquelme@uantof.cl (C.R.)

**Keywords:** eicosapentaenoic fatty acid (EPA; 20:5 n-3), farmed salmon meat, *Nannochloropsis gaditana*, nutritional enhancement, vitamin D_3_, docosapentaenoic fatty acid (DPA; 22:5 n-3), docosahexaenoic fatty acid (DHA, 22:6 n-3)

## Abstract

Omega-3 long-chain polyunsaturated fatty acids (n-3 LC PUFAs) and vitamin D_3_ are essential components of human nutrition. A regular human diet is highly deficient in n-3 LC PUFAs. Fish like salmon are highly recommended in the human diet as they are a major source of high-value n-3 LC PUFAs and vitamin D_3._ The levels of these nutrients have been decreasing over the last few years in farmed salmon, whose production urgently needs sustainable sources of these nutrients. The microalga *Nannochloropsis gaditana* (NG) is known for its naturally high potential for the production of eicosapentaenoic (EPA, 20:5 n-3) fatty acid. A commercial diet for Atlantic salmon was supplemented with 1% and 10% of spray-dried NG grown under controlled conditions for a high EPA content. Salmon were harvested on day 49, following which, boneless and skinless salmon meat was recovered from fish and analyzed for the fatty acid profile, total fat, and vitamin D_3_. Vitamin D_3_, EPA, and docosapentaenoic fatty acid (DPA, 22:5 n-3) levels were significantly increased (*p* < 0.05) by supplementing the basal diet with 10% NG, thus, NG represents a novel, functional, natural ingredient and a sustainable source of n-3 LC-PUFAs that can raise the levels of healthy fats and vitamin D_3_ in farmed salmon meat.

## 1. Introduction

Polyunsaturated fatty acids (PUFAs) are essential components of all cell membranes. They influence membrane fluidity and modulate a wide range of functions in the body [1]. Eicosapentaenoic acid (EPA) and docosahexaenoic acid (DHA, 22:6 n-3) have many health benefits; they are useful against hypertension and Crohn’s disease, and reduce the risk of coronary artery disease and high serum triglycerides [2]. They also have beneficial effects on depression, bipolar disorder, schizophrenia, and dementia [2,3]. n-3 LC PUFAs play an important role in brain function and formation of the structure of the neuronal cell membranes. They are necessary for neurological development, and their tissue concentrations in brain and fetal plasma are dependent on maternal dietary intake, mainly through fatty fish and other seafood consumption [4]. Polyunsaturated fatty acids in the diet are involved in the regulation of cholesterol synthesis by producing a synergistic effect with the dietary cholesterol, which is the main regulator of endogenous cholesterol synthesis [5]. DHA and EPA can reduce the accumulation of cholesterol in the arterial wall as well as lipid content in the visceral adipose tissue and the liver. Lower n-6/EPA+DHA ratio results in lower cholesterol content in the liver [6]. EPA and DHA are produced endogenously by a few fish species and humans, but their biosynthesis is insufficient to meet the physiological demand [7]. α-Linolenic acid (ALA, 18:3n-3) can be converted into EPA and then to DHA, but the conversion, that occurs primarily in the liver, is very limited, with reported rates of less than 15% [8]. Therefore, EPA and DHA fatty acids must be derived from dietary sources [2].

Docosapentaenoic acid (DPA, 22:5 n-3), also known as clupanodonic acid, is an essential n-3 fatty acid found in high amounts in fish oils, and even higher amounts in salmon meat (0.4 g/100 g salmon meat) [9]. DPA is an intermediary between EPA and DHA [10] and may act as a reservoir for EPA and DHA [11]. DPA is an important component of phospholipids found in all animal cell membranes, is involved in the transport and oxidation of cholesterol, and tends to lower plasma cholesterol levels [12,13]. DPA levels are associated with a lower risk of coronary heart disease. It has been shown to reduce the expression of inflammatory genes and is a stronger platelet inhibitor than EPA and DHA [14].

Vitamin D plays an essential role in the growth and maintenance of a healthy skeleton by increasing calcium absorption [15]. Vitamin D deficiency is associated with chronic illnesses, including infectious and cardiovascular diseases, autoimmune disorders, type 2 diabetes [16,17], lethal cancers [17,18] and neurological disorders [3,16,17]. The beneficial health effects of including fish in the human diet have been well documented in several studies. Fatty fish species are dietary sources of n-3 PUFAs [19,20,21,22] and vitamin D_3_ [15,19,23,24]. Vitamin D is present naturally in only a few foods, including oily fish, cod liver oil, and sun-dried mushrooms. Certain countries encouraged the fortification of vitamin D in a few foods, especially milk and butter [16]. For optimum cardiovascular health, the International Society for the Study of Fatty Acids and Lipids recommends a daily intake of 500 mg of n-3 LC-PUFA (EPA+DHA), whereas for individuals with established cardiovascular disease, a daily intake of 1.0 g EPA+DHA is recommended [25]. A high intake of vegetable seed oils and animal products in the western diet leads to high amounts of n-6 fatty acids, resulting in a large increase in the n-6/n-3 ratio and turning the recommended ratio 2–4:1 to about 20–30:1; thus, a greater consumption of food high in n-3 LC-PUFA is needed to correct this nutritional deficiency [26]. Vitamin D deficiency is well-recognized as a world-wide problem for both adults and children. Salmon contains approximately 400 IU (10 µg) of vitamin D_3_/3.5 oz of meat [23]. The US Institute of Medicine estimates the adequate intake (AI) of vitamin D for those with no Sun-mediated synthesis in the skin of 5 µg/day for ages 0–50 years, 10 for 51–70 years and 15 for people over 70 years [27].

Seafood supply from captured fisheries has decreased in the last decade and with the growing demand for seafood for human consumption, aquaculture has become the most important source. Salmon, one of the top seafood products that consumers prefer, is a common farm-raised species. Salmon fish were traditionally fed with high levels of marine sources derived from pelagic fisheries rich in n-3 LC-PUFAs and vitamin D. However, in recent years, there has been a significant decrease in the fishery resource, leading to changes in the composition of farmed salmon meat. Farmed salmon diets have changed from being prepared with 100% marine inputs to the present diets based on 70% plants, which contain low levels of those important nutrients. In Scottish Atlantic farmed salmon, a decrease of up to 50% of EPA+DPA+DHA (g·100 g^–1^) was reported between the years 2006 and 2015 [25]. Consequently, the nutritional content provided to the final human consumer is reduced due to a decrease in the levels of n-3 LC PUFAs [28,29] and vitamin D in farmed salmon [15,28,29].

Microalgae in the aquatic and marine environments are the primary producers of n-3 LC-PUFAs [30,31,32]. Microalgae, owing to their composition and simplicity of cultivation, have great potential in the trend of using natural additives. They are predicted to play important roles as nutritional enhancers in the food industry and as nutraceuticals in the pharmaceutical industry [32]. Algae oils have been recognized as rich sources of EPA and DHA, and have been used in diets for Atlantic salmon [28]. Transgenic camelina expressing algal genes have been tested to produce oil containing n-3 LC-PUFA to replace marine fish oil in salmon feed with no harmful effects on fish performance and no change in the nutritional quality of the meat [33]. However, genetically modified organisms (GMOs) have been linked to causing detrimental effects on human health including horizontal gene transfer [34,35,36] and allergenicity [34,35,37,38], and on animal health with respect to safety concerns on nutritional parameters, digestibility, herbicide and insecticide tolerance [34,35], and chemical composition of GM feed [35].

The urgent need for natural high-quality ingredients will increase with the growth of the aquaculture industry. Therefore, in the future, feed ingredients should be derived from sustainable sources [39]. Among the organisms present in marine and aquatic food webs, algae possess the highest ability to synthesize long-chain PUFAs; unlike, most animals are not able to synthesize essential fatty acids that can be converted into long-chain PUFAs. The fatty acids EPA and arachidonic (20:4 n-6) of the membrane phospholipids are precursors in prostaglandin synthesis. Prostaglandins are precursors to compounds known as tissue hormones [40]. As microalgae are primary producers of EPA and DHA in the marine and aquatic food web, there is increasing interest in their use as additives and supplements in fish feeds [39,41]. The biochemical composition of microalgae can be modulated by altering certain nutrients, environmental stress and culture conditions to induce the microorganisms to produce high concentrations of the desired nutrient [42]. Lipid metabolism is strongly influenced by environmental factors, especially nutrition, and limitation of nitrogen and phosphorus [31]. Although strain- and species-specific variations in fatty acid composition are evident, some microalgae are promising sources of PUFA, especially EPA and DHA [39,43]. The genus *Nannochloropsis* is more widely distributed in the marine ecosystems than that of freshwater ecosystems. *Nannochloropsis* is successfully used as feed in aquaculture due to the high content of long-chain PUFAs, especially eicosapentaenoic acid [40]. *Nannochloropsis gaditana* is a promising marine microalga species for its role in the food web due to its calcium content [44] and its ability to accumulate large amounts of the high-value n-3 fatty acid EPA during nitrogen starvation [45,46]. The aim of this study was to improve the nutritional quality of farmed salmon meat by enhancing the levels of long-chain PUFAs, such as EPA, and vitamin D_3_ by using a natural source like the microalga *Nannochloropsis gaditana* (NG), prepared by spray drying and concentrating NG cultivated under controlled conditions.

## 2. Materials and Methods

### 2.1. NG EPA Induced Growth Conditions

*Nannochloropsis gaditana* strain (Lubián CCMP 527) was maintained under controlled conditions at 20 °C, in 250 mL flasks, with constant aeration at 0.1 *v*/*v*/min with no CO_2_ supply, under constant illumination at 200 µE m^−2^ s^−1^ provided by fluorescent lamps, in “UMA 5” culture medium prepared from fertilizers instead of pure chemicals [47]. The culture medium was prepared using natural seawater and nutrients of analytical grade, and was autoclaved for 15 min. at 121 °C. The strain was sub-cultured every ten days by adding 10% of the old culture medium into 90% of fresh culture medium. The cultures were monitored by microscopic observation using a Leica CME microscope 40X/0.65 to verify the non-occurrence of contamination issues. The culture was scaled up to 1 L using spherical flasks under the same conditions.

*Nannochloropsis gaditana* was cultured (December 2018) in an open raceway type system with a capacity of 14.4 m^3^ under outdoor conditions, grown in batch mode and maintained for 15 days, and subsequently harvested at a dilution rate of 10% per day. Cultures were hatched at 20 °C. A UMA 5 culture medium was used (NaNO_3_: 0.4 g/L, NaH_2_PO_4_: 0.034 g/L, NaHCO_3_: 0.168 g/L and trace elements µmol/L, Zn 0.08, Mn 0.90, Mo 0.030, Co 0.050 and Fe 11.70) [47]. The culture was initiated in a semi-continuous mode, in which 75% of the culture medium and vitamin supplementation (thiamine, biotin, and cyanocobalamin) were deprived. Semi-continuous culturing in outdoor conditions allowed volumetric productivity of 46–56 mg/L/day and 1.6–2.2 g EPA/100 g biomass.

### 2.2. NG Spray Dryer Concentrate

NG algal cells were harvested by centrifugation using a model AS 1936076 continuous centrifuge (GEA Westfalia, Oelde, Germany) at a working flow rate of 2 m^3^/h and a maximum pressure of 3 bar. Once centrifuged, they were dehydrated and concentrated by spray drying (LPG-25 high speed centrifugal spray dryer, (Changzhou Yibu Drying Equipment C0 Ltd., Zhengzhou, Henan, China) using an initial inlet air temperature of 185 °C, a maximum drying chamber temperature of 90 °C and a final temperature of 80 °C for 5–15 s and a working flow rate of 4 L/h.

### 2.3. Nutritional Characterization of NG Concentrate

The proximate analyses, zinc and calcium as well as fatty-acid profiles, were determined using the official methods of analysis of the Association of Official Analytical Chemists (AOAC): crude protein combustion analysis [48] utilizing the calculation 6.25× nitrogen value; sodium and potassium determination [49]; zinc and calcium determination [50]; ash determination [51]; crude fat [52]; moisture content [53]; crude fiber [54] and fatty acid profile [55]. Vitamin D_3_ (colecalciferol) was determined according to EN standard test method [56] All analyses were accredited according to ISO17025. The limit of quantification (LOQ) for vitamin D_3_ was 0.25 µg/100 g.

### 2.4. Experimental Diets

Three different diets were produced: one control diet and two NG supplemented diets (formulated using two different levels of NG: 1% and 10%). NG powder was mixed with the base of a feed formulation in the following proportions to produce three experimental diets: 1.0%, 10.0% and a control diet with no supplemental NG (Table 1). Diets were analyzed for proximate composition sodium, calcium and fatty acid profile according to AOAC standard methods: crude protein combustion analysis [48] utilizing the calculation 6.25× nitrogen value; sodium [49]; calcium [50]; ash determination [51]; crude fat [52,57]; moisture content [53]; crude fiber [54] and fatty acid profile [55]. Vitamin D_3_ (colecalciferol) was determined according to EN standard test method [56] All analyses were accredited according to ISO17025. The limit of quantification (LOQ) for vitamin D_3_ was 0.25 µg/100 g.

### 2.5. Experimental Fish and Feeding

Atlantic salmon (*Salmo salar*) from a single family SNAQ16LSSCO were obtained from AquaGen Chile S.A., Piscicultura Ignao SA, Lago Ranco, Chile). Fish were maintained at 8.6 ± 1 °C, pH 7.11 ± 0.04, 8.51 ± 0.14 dissolved oxygen concentration and 24 h light photoperiod in a flow-through freshwater system in Piscicultura Iculpe-Ilihue, Lago Ranco, Chile. Two hundred and twenty-five fish (104.52 ± 1.29 g each) were distributed randomly in nine tanks (200 L, three tanks per diet). The fish were acclimatized to the tanks for 15 days prior to the start of the trial. The fish were hand-fed 3 mm experimental pellets to satiation twice a day for 49 days (March–May 2018). Environmental parameters (dissolved oxygen concentration, pH and temperature) and feed consumption were measured daily, and fish length and weight were recorded at the beginning and at the end of the experiment. The production parameters feed conversion ratio (FCR) and specific growth rate (SGR) were calculated using the following formulae:

FCR = feed consumed/biomass increase

SGR = 100 × (lnW_2_ − lnW_1_)/feeding days,

where ln is the natural logarithm and W_1_ and W_2_ are the initial and final weights of fish, respectively.

### 2.6. Tissue Sampling

Fifteen fish per treatment (five fish per tank) were randomly sampled on day 59 for proximate, sodium, potassium, and vitamin D_3_ analyses and three fish per tank were randomly sampled for fatty acid profile. The fish were euthanized by cervical dislocation, their liver, gut, and skin were removed, and the obtained meat was weighed, lyophilized in an FDT 8632 model freeze dryer and stored for further analysis. Initial moisture, moisture, and A_w_ (water activity) of the lyophilized meat were determined. Moisture was determined according to AOAC standard method [53]. Aw was determined using a HygroPalm 23-Aw-A digital meter (Rotronic, Hauppauge, NY, USA) All procedures, including handling, treatment, and euthanasia, were performed according to the guidelines provided by the University of Chile animal welfare committee.

### 2.7. Salmon Meat: Proximate, Fatty Acid Profile, Minerals, and Vitamin D Analysis

The proximate analyses, sodium, potassium and fatty-acid profiles, were determined according to AOAC standard methods: proximate analyses: crude protein combustion analysis [48] utilizing the calculation 6.25× nitrogen value; sodium and potassium determination [49]; ash determination [51]; crude fat [52,57]; moisture content [53]; crude fiber [54]; fatty acid profile [55]. Vitamin D_3_ (colecalciferol) was determined according to EN standard test method [56] All analyses were accredited according to ISO17025. The limit of quantification (LOQ) for vitamin D_3_ was 0.25 µg/100 g.

### 2.8. Statistical Analysis

To test for differences in the nutrient composition of salmon farmed meat among the dietary treatment groups, the data were subjected to a one-way analysis of variance (ANOVA) using SPSS Statistics version 25 (IBM Corporation, Armonk, NY, USA). All data were checked for homogeneity of variance prior to the ANOVA. When differences were identified among the groups, multiple comparisons to the control were made using Dunnett’s post-hoc test. The difference was considered significant if *p* was < 0.05. All results are presented as mean ± standard deviation (SD).

## 3. Results

### 3.1. Nutritional Characterization of NG Concentrate

The nutritional characterization of NG concentrate showed high ash, low fiber content, and high Na/K ratio (5.4) in the proximate analysis (Table 2).

The fatty acid profile of NG concentrate showed that myristic acid (14:0), palmitic acid (16:0), palmitoleic acid (9c-16:1), arachidonic [20:4n6], oleic acid (18:1 n-9) and EPA acid (20:5n-3) were the major fatty acids detected, with levels of 7.65, 17.23, 17.93, 3.92, 3.87 and 26.73%, respectively. NG showed a low content of vitamin D_3_ < 0.25 µg/100 g. Analysis of the fatty acid composition of the NG concentrate demonstrated the accumulation of significant levels of EPA (26.73% of total fatty acids), but only a low level of DHA (0.07%) was detected (Table 3).

### 3.2. Fatty Acid and Vitamin D_3_ Composition of the Experimental Diets

The fatty acid profile and vitamin D_3_ content of the experimental diet groups are presented in Table 4. The values of crude fat, myristic fatty acid (14:0), palmitic acid (16:0), palmitoleic acid (9c-16:1), and arachidonic acid (20:4 n-6) increased in both the groups receiving diets supplemented with NG. The value of EPA (20:5 n-3) increased in the group receiving diet supplemented with 10% NG. The control diet group showed higher values of vitamin D_3_ and DHA than the experimental diet groups supplemented with NG (Table 4).

### 3.3. Experimental Fish and Feeding

All experimental diets were received well by the animals, and no pathological or toxic signs were observed. At the end of the forty-nine-day feeding study, no significant differences were observed in feed intake across the three groups. Consumption of the diet supplemented with 10% NG resulted in a significant increase in weight gain and SGR (%) compared to the control diet (Table 5).

### 3.4. Salmon Meat: Proximate, Fatty Acid Profile, Minerals, and Vitamin D

In the lyophilization process, fish from the three dietary treatments had no significant differences in initial moisture and water activity of lyophilized meat. However, the fish treated with 10% NG showed significant differences in the final moisture compared to the control treatment (Table 6).

Proximate analysis of Salmon meat from all dietary treatments showed similar dry matter, ash, protein and lipid compositions, no significant differences were observed (Table 7).

**Figure 1:**
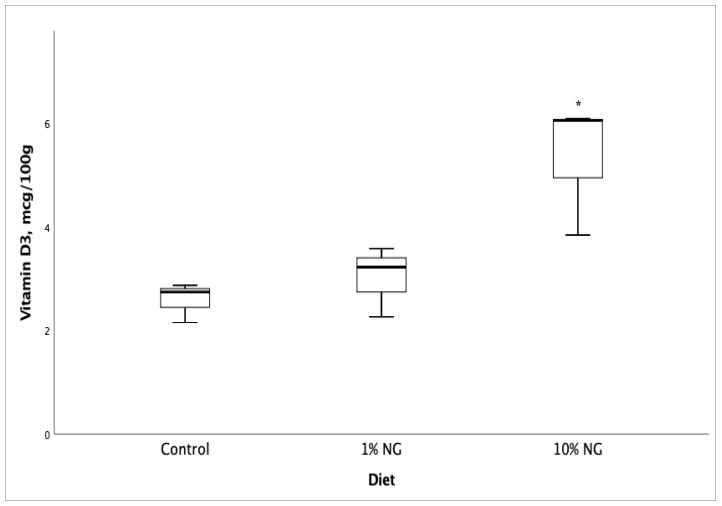
Salmon meat vitamin D_3_ composition after 49 days of feeding the experimental diets. NG = *Nannochloropsis gaditana*, * Significantly different from control (*p* < 0.05) Values are based on the mean ± S.D. n = 5.

**Figure 2:**
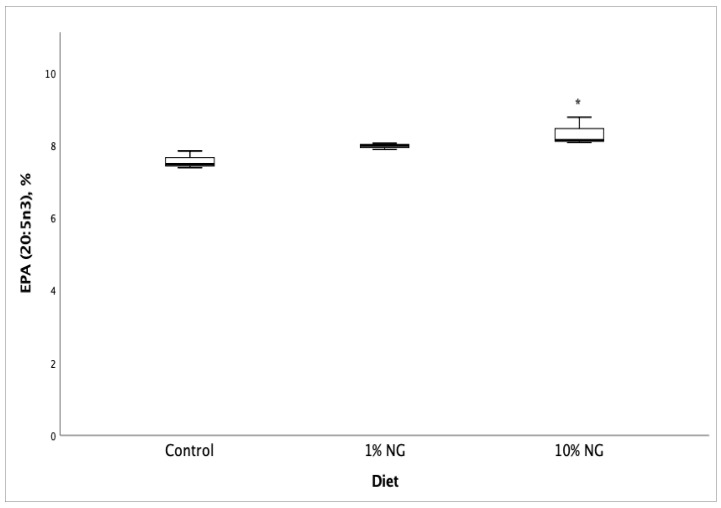
Salmon meat eicosapentaenoic (EPA, 20:5 n-3) fatty acid composition after 49 days of feeding the experimental diets. NG = Nannochloropsis gaditana, * Significantly different from control (*p* < 0.05). Values are based on the mean ± S.D. n = 3.

**Figure 3:**
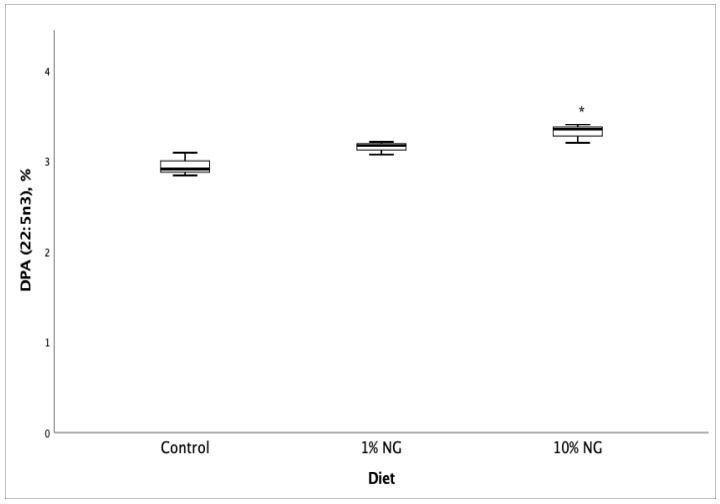
Salmon meat docosapentaenoic (DPA, 22:5 n-3) fatty acid composition after 49 days of feeding the experimental diets. NG = *Nannochloropsis gaditana,* * Significantly different from control (*p* < 0.05). Values are based on the mean ± S.D. n = 3.

Fatty acid profile and vitamin D_3_ analyses of Salmon meat from 10% NG treatment dietary group showed a significant increase (*p* < 0.05) in vitamin D_3_ (Figure 1), EPA (Figure 2), DPA (Figure 3) and the fatty acids palmitoleic (9c-16:1), vaccenic (11c-18:1), C20:1n11 and erucic [22:1n9] when compared to the control dietary group. Stearic (18:0) and oleic (9c-18:1) fatty acids showed a significant decrease. The results showed no significant changes in DHA (22:6 n-3) fatty acid. The fish fed on the 1% NG treatment diet showed significant differences only in palmitic (16:0) and stearic (18:0) fatty acids (Table 8).

## 4. Discussion

Microalgae are a potential source of food and energy due to their high nutritional value and photosynthetic efficiency [58,59]. They are a good source of protein, energy, vitamins, essential fatty acids, pigments, and sterols [58,59,60]. The use of a microalgal strains such as *Spirulina* sp., *Chlorella* sp. or *Scenedesmus* sp. has proven to be a sustainable alternative for the complete replacement of fishmeal in aquaculture [59,61]. Dried microalgae have been used in foods formulated for fish and shrimp as a sustainable substitute for fishmeal protein [58,59]. Cultivation conditions such as CO_2_ concentration, photoperiod, and light intensity favor the production of biomass and lipids in the marine strain *Nannochloropsis* sp. [62,63]. Our study showed that spray-dried NG produced under controlled culture conditions using UMA 5 culture medium is an important source of ash (21.26%), sodium (5.40%), and calcium (4.11%), and a major source of the polyunsaturated fatty acid EPA (26.73% expressed as a percent of total fatty acids). The high EPA values and the absence of DHA obtained with NG treatment are consistent with the values of these fatty acids reported previously for *Nannochloropsis gaditana* [31]. NG concentrate showed no presence of vitamin D_3_. Under natural conditions, planktonic vitamin D accumulates in the marine and aquatic food chain as zooplankton and phytoplankton have high concentrations of D_2_ and D_3_. Fish accumulate large quantities of vitamin D_3_ in their fat tissues, including fat associated with the muscle, but vitamin D_2_ is almost absent in fish tissues. Fish are fully dependent on dietary sources to meet their requirements of vitamin D and do not synthesize this vitamin [64,65]. Although the synthesis of vitamin D_3_ induced by ultraviolet light from 7-dehydrocholesterol (7-DH) has been demonstrated in fish, including rainbow trout, this mode of synthesis does not have a significant contribution (at least for marine fish) in their natural habitat, since most of the UVB irradiation is absorbed in the first few meters of the water column. In rainbow trout, it has been shown that the values of vitamin D metabolites in plasma depend on the environmental concentrations of calcium present in both freshwater and saltwater. Increased environmental calcium is associated with higher transformation to the compound 25,26-dihydroxycholecalciferol, whereas, lower environmental calcium concentrations induce higher conversion to 1,25-dihydroxycholecalciferol-like compound. In Atlantic salmon during the smoltification process and migrating from fresh water to sea water, vitamin D_3_ is regulated by water Calcium (Ca^2+^) concentrations [65]. In our study, vitamin D_3_ levels were increased in Salmon meat enriched with NG. D_3_ concentrations were not detected in the NG concentrate, but nevertheless, NG concentrate showed a high presence of EPA and minerals, with a high content of calcium. This could explain the increase in the concentration of vitamin D_3_ in the treated meat.

Inclusion of the EPA-rich NG concentrate characterized by the presence of 26% EPA in diets for Atlantic salmon had a good effect on fish growth, showed a significant increase in EPA, DPA and a decrease in the n-6/EPA+DHA index, and had a significant effect on fatty acid deposition improving the levels of EPA + DHA (25%) and vitamin D_3_ (106%) in fish meat, thus, ultimately resulting in an improvement in the nutritional quality of fish meat for human consumption (Table 9).

## 5. Conclusions

This study has shown that the inclusion of dietary spray-dried NG concentrate in the fish diet resulted in enhancement of EPA (20:5 n-3), DPA (22:5 n-3), and vitamin D_3_ levels of salmon farmed meat without compromising the feed conversion rate or fish growth. Increasing these bioactive compounds could help attenuate or prevent diseases or disorders associated with its deficiency. The dietary value of salmon farmed meat is effectively improved by spray-dried NG concentrate prepared from non-GMO *Nannochloropsis gaditana* grown under controlled culture conditions to include health benefits for consumer populations.

## Figures and Tables

**Table 1:** Formulation, proximate, sodium and calcium composition of experimental diets.

Ingredients	(%)		
Fish meal	28.00	28.00	28.00
Soybean concentrate	16.64	15.64	14.90
Whole wheat	14.00	14.00	
Wheat meal			7.00
Fish oil	19.7	19.7	18.4
Corn gluten	10.00	10.00	10.00
Chicken viscera meal	10.00	10.00	10.00
Monocalcium phosphate	1.00	1.00	1.00
Mineral vitamin premix	0.38	0.38	0.38
L-Lysine	0.21	0.21	0.27
DL-Methionine	0.07	0.07	0.08
*Nannochloropsis gaditana* (NG)		1.00	10.00
Total	100.00	100.00	100.00
**Chemical composition of diets**	**(%)**		
Lipids, % dm	19.54	19.61	19.66
Protein, % dm	45.76	45.96	44.38
Fiber, % dm	1.35	1.11	1.38
Ash, % dm	9.84	10.03	11.08
Moisture, %	6.68	6.28	6.54
Calcium %	1.10	1.11	1.21
Sodium %	0.38	0.40	1.08

NG = *Nannochloropsis gaditana* concentrate, dm = dry matter basis.

**Table 2:** Chemical composition of NG concentrate.

Header	Header
Crude fat, %	4.18
* Protein, %	36.54
Crude fiber, %	0.01
Ash, %	21.26
Moisture, %	4.44
Sodium. %	5.40
Potassium. %	1.00
Zinc ppm	171
Calcium. %	4.11

* Percentage N × 6.25. %, g per 100 g of sample.

**Table 3:** Fatty acid composition of NG concentrate.

Header	Header
Vitamin D3 µg/100 g	<0.25
Fatty acid profile (*expressed as percent of total fatty acids*)	
Myristic (14:0)	7.65
Myristoleic (9c-14:1)	0.16
C15:0	0.28
Palmitic (16:0)	17.23
Palmitoleic (9c-16:1)	17.93
Stearic (18:0)	0.33
Elaidic (9t-18:1)	0.19
Oleic (9c-18:1)	3.87
Vaccenic (11c-18:1)	0.37
Linoleic (18:2n6)	3.17
**Linolenic (18:3n3)**	**0.17**
g-Linolenic [C18:3n6]	0.41
Gonodic (20:1n9)	0.05
C20:2	0.18
Arachidonic [20:4n6]	3.92
**EPA (20:5n3)**	**26.73**
C22:2n6	0.08
**DPA (22:5 n-3)**	**0.00**
**DHA (22:6n3)**	**0.07**
Lignoceric (24:0)	0.20

n-3 fatty acid values are in bold.

**Table 4:** Fatty acid and vitamin D_3_ composition of experimental diets.

	Control	1% NG	10% NG
Vitamin D3 µg/100 g	15.40 ± 4.0	13.40 ± 3.50	12.80 ± 3.30
Crude Fat, %	19.54	19.61	19.66
	Fatty acid profile (*expressed as percent of total fatty acids*)		
Myristic (14:0)	6.21	6.23	6.32
Myristoleic (9c-14:1)	0.06	0.05	0.06
C15:0	0.47	0.47	0.47
C15:1n5	0.02	0.01	0.02
Palmitic (16:0)	17.51	17.86	17.75
Palmitoleic (9c-16:1)	7.13	7.21	7.60
Margaric (17:0)	0.39	0.40	0.39
10c-17:1	0.27	0.26	0.27
Stearic (18:0)	3.71	3.80	3.66
Elaidic (9t-18:1)	0.18	0.18	0.19
Oleic (9c-18:1)	12.43	12.56	12.25
Vaccenic (11c-18:1)	3.18	3.26	3.09
Linoelaidic (18:2t)	0.00	0.00	0.00
Linoleic (18:2n6)	4.30	4.34	4.31
**Linolenic (18:3n3)**	**1.05**	**1.05**	**1.00**
g-Linolenic [C18:3n6]	0.21	0.21	0.22
**Stearidonic (18:4n3)**	**2.28**	**2.22**	**2.16**
Arachidic (20:0)	0.34	0.35	0.33
Gonodic (20:1n9)	1.70	1.71	1.60
C20:2	0.00	0.00	0.00
**Homo-α-linolenic(20:3n3)**	**0.08**	**0.08**	**0.09**
Arachidonic [20:4n6]	0.70	0.74	0.88
**3n-Arachidonic (20:4n3)**	**0.00**	**0.00**	**0.00**
**EPA (20:5n3)**	**12.52**	**12.51**	**12.86**
Behenoic (22:0)	0.16	0.17	0.17
Erucic [22:1n9]	0.34	0.34	0.33
C22:2n6	0.59	0.62	0.58
Adrenic [C22:4n6]	0.09	0.09	0.11
**DPA (22:5 n-3)**	**1.62**	**1.62**	**1.53**
**DHA (22:6n3)**	**6.73**	**6.60**	**6.31**
Lignoceric (24:0)	0.10	0.09	0.10
Nervonic (24:1n9)	0.44	0.46	0.45

n-3 fatty acid values are in bold.

**Table 5:** Effect of NG concentrate inclusion level on weight-gain, Specific growth rate-SGR and Feed conversion ratio-FCR animals feed experimental diets.

	Weight Gain (g)	SGR (%)	Feed Conversion Ratio-FCR
Control	102.95 ± 1.4	1.39 ± 0.01	1.11 ± 0.03
1% *NG*	113.97 ± 8.03	1.49 ± 0.09	1.45 ± 0.24
10% *NG*	122.04 ± 1.3 *	1.59 ± 0.01 *	0.93 ± 0.26

NG = *Nannochloropsis gaditana*, * Significantly different from control (*p* < 0.05). Values are based on the mean ± S.D. of three replicate tanks of 20 fish; n = 3.

**Table 6:** Effect of NG inclusion on Initial moisture, final moisture and Aw of lyophilized Salmon meat enriched with *Nannochloropsis gaditana*.

	Initial Moisture	Final Moisture	A_w_
Control	74.84 ± 0.11	1.15 ± 0.82	0.113 ± 0.03
1% *NG*	75.33 ± 0.99	0.67 ± 0.43	0.106 ± 0.01
10% *NG*	74.54 ± 0.41	2.93 ± 0.59 *	0.109 ± 0.01

NG = *Nannochloropsis gaditana,* * Significantly different from control (*p* < 0.05). Values are based on the mean ± S.D. n = 5.

**Table 7:** Salmon meat proximate, sodium and potassium composition after 49 days of feeding the experimental diets.

	Control	1% NG	10% NG
Moisture, %	74.84 ± 0.17	75.33 ± 0.99	74.54 ± 0.39
Crude fat, %	4.22 ± 0.69	3.61 ± 0.24	4.30 ± 0.74
Protein, % *	19.07 ± 0.61	19.33 ± 0.80	19.21 ± 0.78
Ash, %	2.49 ± 0.18	2.57 ± 0.19	2.55 ± 0.20
Sodium, ppm	942.00 ± 76	900.33 ± 31.00	965.00 ± 69.46
Potassium, %	1.75 ± 0.06	1.72 ± 0.04	1.70 ± 0.06

* Crude protein = %N × 6.25, ppm = parts per million. NG = *Nannochloropsis gaditana*, Values are based on the mean ± S.D. n = 5.

**Table 8:** Salmon meat fatty acid and vitamin D_3_ composition after 49 days of feeding the experimental diets.

	Control	1% NG	10% NG
Vitamin D_3_ µg/100 g	2.58 ± 0.38	3.02 ± 0.68	5.32 ± 1.28 *
Crude Fat, %	4.22 ± 0.69	3.61 ± 0.24	4.30 ± 0.74
Fatty Acid Profile *(expressed as percent of total fatty acids)*			
Myristic (14:0)	4.42 ± 0.07	4.35 ± 0.04	4.49 ± 0.08
Myristoleic (9c-14:1)	0.04 ± 0.00	0.04 ± 0.00	0.04 ± 0.00
C15:0	0.35 ± 0.00	0.34 ± 0.00	0.35 ± 0.00
C15:1n5	0.00	0.00	0.00
Palmitic (16:0)	15.69 ± 0.16	15.15 ± 0.09 *	15.29 ± 0.28
Palmitoleic (9c-16:1)	6.00 ± 0.11	6.01 ± 0.01	6.26 ± 0.10 *
Margaric (17:0)	0.32 ± 0.00	0.30 ± 0.00	0.30 ± 0.01
10c-17:1	0.28 ± 0.07	0.20 ± 0.00	0.28 ± 0.06
Stearic (18:0)	4.03 ± 0.03	3.90 ± 0.04 *	3.80 ± 0.02 *
Elaidic (9t-18:1)	0.17 ± 0.00	0.18 ± 0.00	0.18 ± 0.00
Oleic (9c-18:1)	16.85 ± 1.01	15.99 ± 0.09	15.63 ± 0.46 *
Vaccenic (11c-18:1)	3.57 ± 0.03	3.61 ± 0.03	3.67 ± 0.01 *
Linoelaidic (18:2t)	0.00	0.00	0.00
Linoleic (18:2n6)	6.71 ± 0.38	6.38 ± 0.08	6.16 ± 0.15
**Linolenic (18:3n3)**	**1.33 ± 0.08**	**1.29 ± 0.02**	**1.24 ± 0.02**
g-Linolenic [C18:3n6]	0.20 ± 0.01	0.20 ± 0.00	0.18 ± 0.00
**Stearidonic (18:4n3)**	**1.67 ± 0.04**	**1.71 ± 0.01**	**1.74 ± 0.01**
Arachidic (20:0)	0.25 ± 0.00	0.24 ± 0.01	0.30 ± 0.08
Gonodic (20:1n9)	1.61 ± 0.02	1.60 ± 0.02	1.63 ± 0.04
C20:1n11	1.24 ± 0.06	1.34 ± 0.02	1.56 ± 0.01 *
C20:2	0.00	0.00	0.00
**Homo-α-linolenic(20:3n3)**	**0.14 ± 0.00**	**0.13 ± 0.00**	**0.14 ± 0.00**
Arachidonic [20:4n6]	0.83 ± 0.02	0.83 ± 0.01	0.81 ± 0.03
**3n-Arachidonic (20:4n3)**	**0.00**	**0.00**	**0.00**
**EPA (20:5n3)**	**7.56 ± 0.24**	**7.94 ± 0.09**	8.33 ± 0.38 *
Behenoic (22:0)	0.13 ± 0.00	0.13 ± 0.00	0.12 ± 0.01
Erucic [22:1n9]	0.27 ± 0.00	0.28 ± 0.00	0.29 ± 0.00 *
C22:1n11	1.62 ± 0.08	1.70 ± 0.02	1.91 ± 0.07 *
C22:2n6	0.90 ± 0.02	0.97 ± 0.03	1.06 ± 0.04
Adrenic [C22:4n6]	0.00	0.00	0.00
**DPA (22:5 n-3)**	**2.95 ± 0.13**	**3.15 ± 0.07**	3.31 ± 0.10 *
**DHA (22:6n3)**	**11.87 ± 0.89**	**12.85 ± 0.74**	**11.83 ± 0.39**
Lignoceric (24:0)	0.11 ± 0.00	0.10 ± 0.00	0.10 ± 0.00
Nervonic (24:1n9)	0.47 ± 0.01	0.46 ± 0.00	0.47 ± 0.00
Total n3	25.52 ± 1.13	27.06± 0.56	26.60± 0.79
Total n6	8.64 ± 0.34	8.38 ± 0.11	8.21 ± 0.14
n6/EPA+DHA	0.44 ± 0.03	0.40 ± 0.01	0.40 ± 0.01

n-3 fatty acids are in bold type. NG = *Nannochloropsis gaditana*, * Significantly different from control (*p* < 0.05). Values are based on the mean ± S.D. n = 5 for vitamin D_3_ and n = 3 for fatty acid profile.

**Table 9:** Recommended daily intake of n-3 Fatty acid and vitamin D_3_ compared with n-3 Fatty acid and vitamin D_3_ composition of experimental farmed salmon meat.

	EFSA ^1^	ISSFAL ^2^	NIH ^3^	Salmon Meat Not Enriched with NG Concentrate	Salmon Meat Enriched with 1% NG Concentrate	Salmon Meat Enriched with 10% NG Concentrate
EPA + DHA per day	250 mg ^5^	500 mg ^6^	250 mg	693 mg/100 g	750.51 mg/100 g	866.69 mg/100 g
Vitamin D_3_ RDA ^4^, age 0–1 years age 1–70 years and > 70 years	10 µg 15 µg 15 µg		15 µg 20 µg	2.58 µg/100 g	3.02 µg/100 g	5.32 µg/100 g

^1^ EFSA = European Food Safety Authority https://www.efsa.europa.eu/. ^2^ ISSFAL = International Society for the study of Fatty Acids and Lipids [66] https://www.issfal.org/. ^3^ NIH = National Institutes of Health, 2015–2020 *Dietary Guidelines for Americans*, “For the general population, associated with reduced cardiac deaths among individuals with and without preexisting CVD” [67]. ^4^ RDA = Recommended Dietary Allowance: Average daily level of intake sufficient to meet the nutrient requirements of nearly all (97–98%) healthy individuals; often used to plan nutritionally adequate diets for individuals [68]. ^5^ Mg EPA + DHA per day adults should be consuming through oily fish consumption. ^6^ Minimum daily intake for cardiovascular health.
